# Complex Wide-necked and Lobulated Aneurysm of the Middle Cerebral Artery Bifurcation: Treatment with a pCONUS2 Neck Bridging Device and p48MW Flow Modulation Device

**DOI:** 10.1007/s00062-019-00862-5

**Published:** 2019-12-05

**Authors:** Alexander Sirakov, Marta Aguilar-Perez, Muhammad AlMatter, Hans Henkes

**Affiliations:** 1 Radiology Department, UH St Ivan Rilski, Sofia, Bulgaria; 2 Neuroradiologische Klinik, Neurozentrum, Klinikum Stuttgart, Stuttgart, Germany; 3 Medizinische Fakultät, Universität Duisburg-Essen, Essen, Germany

## Introduction

With the recent improvement of the techniques, armamentarium and operator skills, a growing number of intracranial aneurysms can be endovascularly treated; however, some aneurysms, particularly those located on arterial bifurcations, remain a challenge for the traditional endovascular techniques and demand a more complex approach [1]. Balloon remodeling and stent-assisted coiling have limitations while being applied in the case of complex anatomy [2]. More recently, dedicated devices such as the pCONUS (phenox, Bochum, Germany), the PulseRider (Pulsar Vascular, Los Gatos, CA, USA) and the eCLIPs (Evasc Medical Systems, Vancouver, Canada) have entered the market. These devices share the common feature of providing extra coverage at the aneurysm neck to prevent coil prolapse into the parent vessel. Endoluminal flow diversion (FD) appears to be a straightforward solution for complex and unfavorable anatomy with good angiographic results and acceptable complication rates; however, the fate of the covered branches and the delayed aneurysm occlusion are still a major concern [3].

We present a case where the combination of a pCONUS2 neck-bridging device and p48MW Flow Modulation Device were used for the treatment of a complex, wide-necked aneurysm of the right middle cerebral artery (MCA).

## Case Report

A 72-year-old female patient was admitted for treatment of an incidentally found aneurysm of the right MCA. Magnetic resonance imaging (MRI) and digital subtraction angiography (DSA) examinations confirmed the MCA aneurysm measuring 5.5 × 5 mm with a neck width of 5.7 mm. Aneurysmal irregularities with a secondary lobule adjacent to the inferior trunk was also noted on the rotational angiography and 3D reconstructions. The goal of the treatment was the prevention of a future rupture of the aneurysm. The lobulated irregularities of the aneurysmal sac and the wide neck were a major concern. Microsurgical clipping was recommended to and declined by the patient but pCONUS-assisted coiling was considered feasible. The intended treatment, together with potential alternatives, respective chances, and risks were explained to the patient. Informed consent was obtained in written form.

**Fig. 1:**
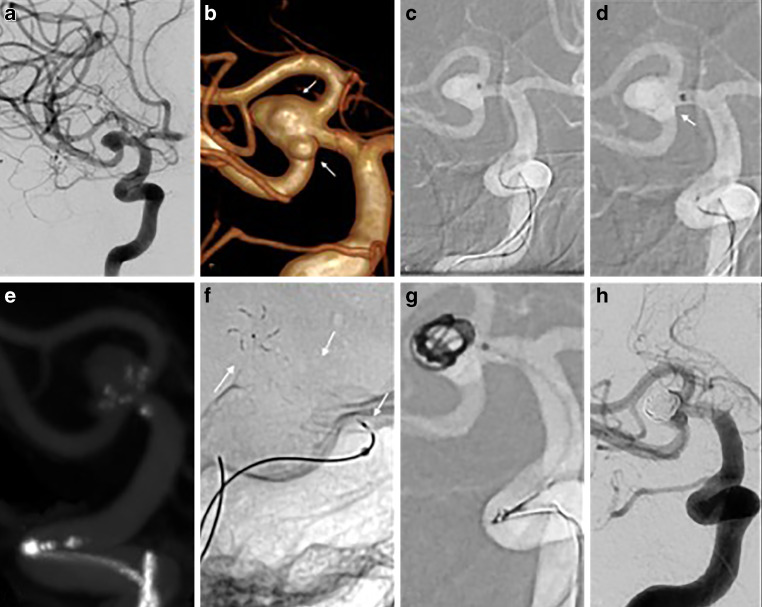
First stage of the endovascular treatment of a complex middle cerebral artery (MCA) bifurcation aneurysm. The angiographic working projection (left anterior oblique **a**) shows the anatomical details of the aneurysm. Rotational digital subtraction angiography (DSA) with 3D reconstruction (**b**) confirmed the presence of focal irregularities (*superior arrow*) and a secondary lobule (*inferior arrow*) adjacent to the inferior trunk, together with the unfavorable dome-to-neck ratio. Catheterization of the aneurysmal sac was carried out under road map guidance (**c**). The crown of the pCONUS2 device was deployed with the petals placed across the aneurysmal neck (*arrow* in **d**, **e**). Note the radiopacity of the device (*arrows* at the level of the crown, distal and proximal markers) as shown in this single shot fluoroscopic image (**f**). The petals of the device (**g**) provided enough protection for both the neck and the branches while coiling the aneurysm. A final DSA run at the end of the procedure (**h**), confirmed the sufficient occlusion of the aneurysmal sac. The small lobule adjacent to the inferior trunk of the MCA was still present at the final DSA run

With the patient under general anesthesia and using a standard right common femoral approach, an 8 Fr Softip guide catheter (Boston Scientific, Marlborough, MA, USA) was tracked into the right internal carotid artery. Initially, the aneurysm was catheterized using a Prowler Select Plus microcatheter (Cerenovus, Irvine, CA, USA), and a pCONUS2 4–15–6 mm was then deployed but not detached. A SL10 microcatheter (Stryker Neurovascular, Fremont, CA, USA) was then navigated inside the aneurysm though the pCONUS2 and subsequent coiling was initiated; however, due to the dense coil packing inside the main sac of the aneurysm, the secondary lobule adjacent to the inferior trunk remained untreated. Neither coil protrusion nor evidence of thrombus formation were observed on the final angiography. Finally, the pCONUS2 was electrolytically detached (Fig. 1).

Follow-up angiography performed 2 months later showed stable occlusion of the main aneurysmal sac with persistent perfusion of the lobule adjacent to the inferior trunk. In this situation, there was little reason to assume that the rupture risk of this aneurysm had been completely eliminated by the first treatment, as the literature suggests that irregular surface and daughter sacs are associated with a higher risk of rupture [4]. The size of the lobule was also a matter of concern as a conservative approach is usually advocated for aneurysms below 5 mm in diameter; however, in clinical practice patients with aneurysmal subarachnoid hemorrhage (aSAH) in small aneurysms are routinely observed. Bender et al. [5] observed in a 25-year study including 1306 patients with an aSAH that the majority and increasing proportion of ruptured aneurysm were small or very small with an increase in the percentage of aneurysms <5 mm from 29% to 50%. These data together with the fact that older patients have a higher risk of unfavorable clinical outcome after aSAH [6] were explained in detail to the patient and relatives. A second treatment was accordingly offered to and accepted by the patient. Due to the presence of the pCONUS2 device a microsurgical option was rejected. An endovascular-endosaccular option was not appealing due to the small size and the sharp angle with the parent vessel. Instead, endoluminal FD offered a good risk/benefit ratio according to our experience. Currently available studies also indicated that these devices may have higher benefits due to lower rates of aneurysm recurrence rate compared to both simple coiling and stent-assisted techniques [7, 8].

**Fig. 2:**
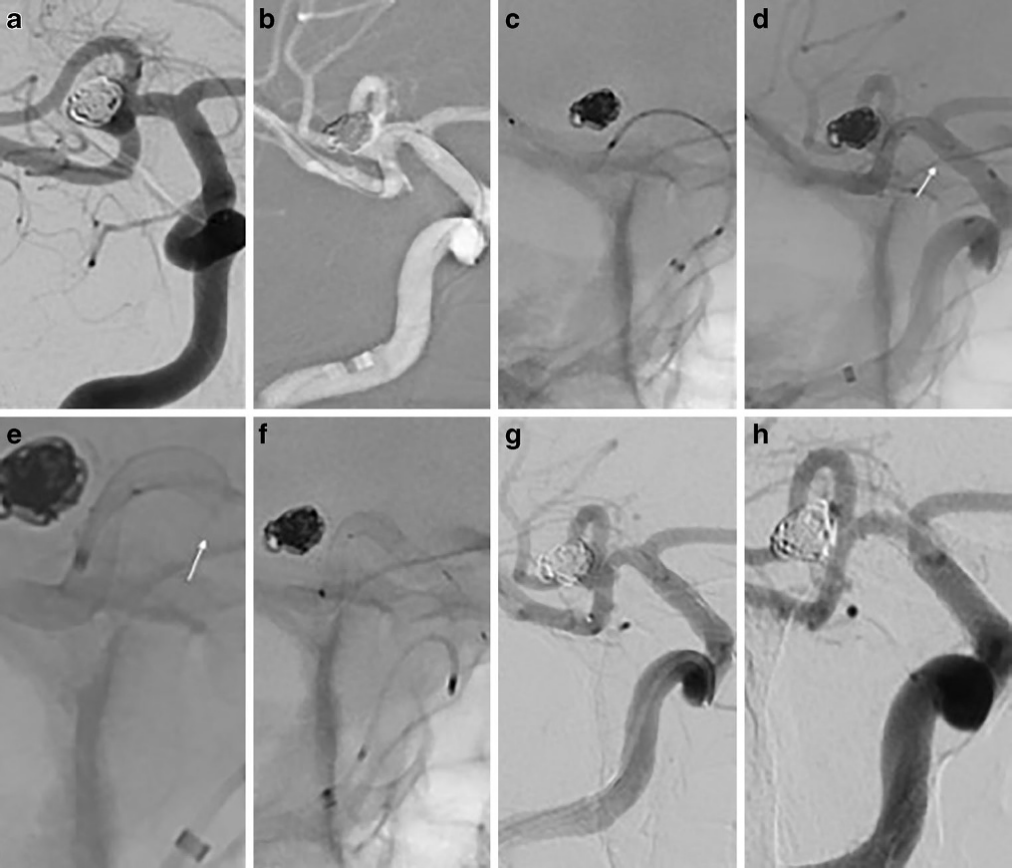
Second and final stage of the endovascular treatment of a complex MCA bifurcation aneurysm. Working projection showing stable occlusion of the main aneurysmal sac with persistent perfusion of the lobule adjacent to the inferior trunk (**a**). Distal and straightforward navigation (**b**) of the microcatheter used for the p48MW deployment was achieved. The first low-profile flow modulation device p48MW 3/18 was carefully loaded and positioned inside the microcatheter at the level of the right MCA (**c**), following by complete deployment of the FD (**d**), which expanded properly inside the crown of the pCONUS2 device with slight “fish mouth” appearance proximally (**d**, *arrow*). Note the partial resolution of the “fish mouth” while applying only gentle massage with the microcatheter (**e**, *arrow*). The implantation of the second p48MW (3/12) completely resolved the proximal narrowing of the first FD (**f**), while adequate vessel apposition was achieved. A final DSA run at the end of the procedure confirmed the patency of both flow diverters and the parent arteries (**g**). The 12-month follow-up imaging (**h**) demonstrated the complete and definitive occlusion of the target aneurysm. Note the patency of the implanted pCONUS2 device and both p48MW

In a second treatment session an access to the aneurysm was again achieved via the right ICA. The inferior trunk of the right MCA was catheterized and a Prowler Select Plus (Cerenovus) was tracked distally through the pCONUS2. Subsequently, a 3 mm × 18 mm p48MW was carefully deployed into the inferior trunk and proximally in the right M1 segment. Correct and complete expansion of the p48MW was confirmed under pulsed fluoroscopy. After total deployment, the proximal portion of the device showed a slight “fish mouth” appearance with incomplete wall apposition. Navigation with the microcatheter through the p48MW partially resolved the proximal collapse. In order to avoid any potential problem, a second p48MW (3 mm × 12 mm) was implanted proximally in an overlapping way. The final DSA run demonstrated the patency of all branches with no thromboembolic complications (Fig. 2).

Both procedures were well tolerated by the patient, and the neurological status was unchanged from baseline each time. Follow-up angiography at 12 months showed complete exclusion of the aneurysm from the circulation, including the small lobule adjacent to the inferior trunk, and patency of all branches, including the jailed superior trunk.

## Discussion

Dangerous aneurysmal flow changes and related hemodynamic patterns may result in shape changes of the aneurysmal sac. The presence of a number of geometric features, such as irregularities, focal bulging or lobulation, are considered as predictors of aneurysmal rupture [9, 10]. Despite the plethora of available endovascular techniques, such as balloon remodeling [11], stent-assisted coiling [12] or waffle-cone technique [13] treating aneurysms with unfavorable dome-to-neck ratio and/or incorporated efferent branches remains challenging to the neurointerventionalist [14]. Recently, several devices aiming specifically at dealing with these aneurysms have been introduced, including the PulseRider [15], eCLIPs [16], and pCONUS [17] devices. These devices share the common feature of providing extra coverage at the aneurysm neck to prevent coil prolapse into the parent vessel. Intrasaccular flow disruptors, such as the WEB (MicroVention, Aliso Viejo, CA, USA) present also an alternative for the treatment of wide-necked aneurysms [18]. Flow diversion is being increasingly used to treat bifurcation aneurysms. In cases of MCA bifurcation aneurysms, FD treatment is feasible with good angiographic results and acceptable complication rates; however, compared to the results of other endovascular techniques and to surgery, total occlusion seems to be less frequent [3, 19, 20]. Most of these new tools available on the market target the temporary or permanent reconstruction of the aneurysmal neck [21, 22], the parent artery or both but in certain scenarios, alternative or combination techniques are mandatory to secure the patency of the incorporated branches and deal with the troublesome anatomy.

This case represents a situation where a pCONUS2 device [23] was successfully used to protect the incorporated efferent branches in a MCA bifurcation aneurysm during coiling. Secondly, the implantation of the p48MW low profile FD [24] over the neck and across the pCONUS2 resulted in a significant hemodynamic change, reconstructing the MCA bifurcation and redirecting the blood flow away from the aneurysm in the intended fashion.

**Fig. 3:**
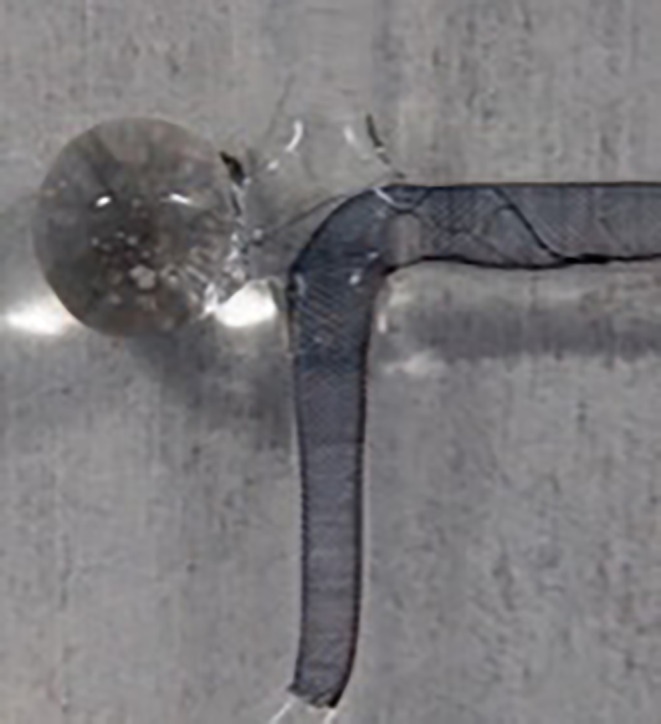
Glass model of an MCA bifurcation aneurysm showing a pCONUS2 bifurcation device implanted with its petals at the level of the “aneurysmal” neck. A p48MW 2/15 mm is deployed through the pCONUS2 from the “inferior MCA trunk” to the “M1 segment”. There is only a small amount of metal connecting the six petals with the shaft of the p48. This allows the uncompromised expansion of the flow diverter

The special design of the pCONUS2 allow the combination with the p48MW while providing the same coil retention that the pCONUS1 offers (Fig. 3). No technical difficulties were encountered during microcatheter navigation through the pCONUS2. The radial force of the p48MW allowed adequate wall apposition of the device during deployment.

It is believed that the combined use of the pCONUS2 for assisted coil occlusion together with an endoluminal FD, such as p48MW, allows an improved treatment for selected, complex bifurcation aneurysms; however, a true bifurcation flow diverter might have the potential to facilitate this kind of treatment even more.
